# Landscape Variation in Tree Species Richness in Northern Iran Forests

**DOI:** 10.1371/journal.pone.0121172

**Published:** 2015-04-07

**Authors:** Charles P.-A. Bourque, Mahmoud Bayat

**Affiliations:** 1 Faculty of Forestry and Environmental Management, University of New Brunswick, New Brunswick, Canada; 2 Department of Forestry, Natural Resources Faculty, University of Tehran, Karaj, Iran; University of Calgary, CANADA

## Abstract

Mapping landscape variation in tree species richness (SR) is essential to the long term management and conservation of forest ecosystems. The current study examines the prospect of mapping field assessments of SR in a high-elevation, deciduous forest in northern Iran as a function of 16 biophysical variables representative of the area’s unique physiography, including topography and coastal placement, biophysical environment, and forests. Basic to this study is the development of moderate-resolution biophysical surfaces and associated plot-estimates for 202 permanent sampling plots. The biophysical variables include: (i) three topographic variables generated directly from the area’s digital terrain model; (ii) four ecophysiologically-relevant variables derived from process models or from first principles; and (iii) seven variables of Landsat-8-acquired surface reflectance and two, of surface radiance. With symbolic regression, it was shown that only four of the 16 variables were needed to explain 85% of observed plot-level variation in SR (i.e., wind velocity, surface reflectance of blue light, and topographic wetness indices representative of soil water content), yielding mean-absolute and root-mean-squared error of 0.50 and 0.78, respectively. Overall, localised calculations of wind velocity and surface reflectance of blue light explained about 63% of observed variation in SR, with wind velocity accounting for 51% of that variation. The remaining 22% was explained by linear combinations of soil-water-related topographic indices and associated thresholds. In general, SR and diversity tended to be greatest for plots dominated by *Carpinus betulus* (involving ≥ 33% of all trees in a plot), than by *Fagus orientalis* (median difference of one species). This study provides a significant step towards describing landscape variation in SR as a function of modelled and satellite-based information and symbolic regression. Methods in this study are sufficiently general to be applicable to the characterisation of SR in other forested regions of the world, providing plot-scale data are available for model generation.

## Introduction

As species and species habitat continue to be eliminated from the global landscape, delineation of tree species distribution, species richness and diversity, and changes in associated distributions is fundamental to conservation planning and management [1, 2]. Spatiotemporal variation in abiotic variables, such as in incident solar radiation, near-surface atmospheric temperature, and soil water and nutrient content, defines the long term division of plant species and their associated abundance in plant communities within landscapes [3–5]. The precise combination of abiotic variables driving variation in local tree species richness (SR; number of species per unit area) invariably differs across ecosystems; e.g., in closed-canopy forests of the tropics, the hydrological network and height above nearest drainage were shown to serve as reasonable models of spatial organisation in the area’s forests [6,7], whereas in temperate, mountain, and coastal forests, prevailing wind and thermal regimes were considered to be more important with respect to forest structure and diversity [3,5,8]. Consequently, assessing the relative importance of environmental factors to local variation in SR is essential to understanding the underlying ecological mechanisms affecting regional trends in this variable [6] at scales relevant to land managers (i.e., 30 m to 2 km; [9]).

Characterising SR and environmental variables over large areas is often very difficult, to impossible, to achieve with field measurements alone [10]. Publically-funded remote sensing (RS) platforms and their products offer an affordable, nearly continuous means of observing the earth’s surface from reflectance and thermal signatures at coarse to medium spatiotemporal and spectral resolutions [1,10].

Variables related to species diversity and SR, like vegetation indices and within-image spectral heterogeneity have been used in the past to map species diversity and richness at medium spatial resolutions, attaining accuracies of 40–80% [2]. Assessing environmental variables at mid-resolutions (< 100 m), such as local windiness, however, is impossible with RS methods. To assist with defining the biophysical attributes of landscapes and/or their surrogate variables (e.g., slope, slope orientation, wetness index), terrain analysis and process models have been widely used to generate the required site information inaccessible by RS and field surveys [11–13]. Analyses relating SR, tree-growth, plot-level, or photo-interpreted forest-cover descriptions to modelled abiotic quantities or their surrogate values include those of Byun et al. (2013), Ashraf et al. (2012, 2013), Lebourgeois et al. (2005), and Austin et al. (1996) [14–18]. Level of agreement between modelled and observed data generally varies with the spatial resolution of the forest-sampling data and digital terrain model (DTM) used in the analysis [15].

In this study, we develop numerical surfaces depicting the physical environment for a high-elevation deciduous forest. Spatial pattern in a onetime survey of plot SR (2003) is examined relative to spatial variation in plot-estimates of 16 different site variables, including seasonal insolation, mean air temperature (${\bar{\text{T}}}_{a}$), height above nearest drainage point (HNDP), topographic wetness index (TWI), relative humidity (RH), wind velocity, and surface reflectance and radiance. Many of these environmental variables, either directly or through their proxies, have been shown to correlate fairly well with SR [3]. The objective of the research is to determine from the list of derived variables in Table 1, which variables contribute the most to a rational explanation of observed variation in SR.

**Table 1:** Sixteen variables considered in the analysis of tree species richness in the Kheyrud Forest of northern Iran.

Variable	Derivation and/or source	Comments
**Slope** (°)	Slope can be estimated directly from finite-difference evaluations of DTM-height data with GIS; the 10-m resolution DTM is based on a bi-cubic interpolation of the 30-m GDEM v. 2 (Fig 1a)	Slope is used in this study as an indicator for the potential of mass wasting occurring in steep terrain. Catastrophic slope failures can lead to sizeable debris flows and landslides that can accentuate local within-site heterogeneity and promote species proliferation during site recovery [19,20]
**Height above nearest drainage point** (HNDP; m)	Based on algorithms described in Murphy et al. and Rennó et al. [11,21]	HNDP (Fig 1b) provides a simple measure of potential drainage [7] and is described as the vertical separation between raised dry land and a localised estimate of the water-table level based on surface water. For a particular “dry” cell, the “wet” cell nearest its located by means of an iterative search function that minimises the horizontal distance between the subject “dry” cell and the “wet” cells downslope, while adhering to DTM flow directions and pathways [11,21]; HNDP = 0 m signifies visible surface water, while large HNDP’s signify low water tables and potentially dry soil conditions
**Topographic wetness index** (TWI; non-dimensional)	Based on TWI = ln(A_s_/tan(β)), where As is the specific contributing area and tan(β) is the slope along the flow direction (o; [22])	Topography redistributes precipitation and soil water and, as a result, surfaces of TWI (Fig 1c) can be developed from DTM-height data alone [23]. Methods of computing TWI vary in the way As is calculated [24]; here, we use the mass-flux method of Peckham [25]
**Growing-season accumulated potential solar radiation** (MJ m^-2^)	Derived from LanDSET-model calculations [26,27]; ArcGIS has the capacity to generate similar surfaces	Solar radiation (Fig 1d) has the potential of altering tree growth and tree distribution differently for different species. Incoming solar radiation is evaluated here as a function of (i) DTM-based calculations of slope, slope orientation, view factor, horizon angle, and terrain configuration factors, (ii) sun-earth geometry and solar-illumination angles, and (iii) solar-flux calculations at the top of the atmosphere
**Air Temperature** (°C)	Vertical variation in temperature is based on an assumed environmental temperature lapse rate of 6.5°C km^-1^ [28] and initial surface mean air temperature of 21.2°C at Noushahr climate station	Physiological variable (Fig 1e) associated with plant photosynthesis, transpiration, metabolism, and growth; plant species differ in their response to atmospheric temperatures and associated accumulated seasonal heating [27]
**Relative humidity** (RH, %)	Determined in the same way as described in Bourque & Matin (2012) [12]; consult S1 Appendix	Physiological variable (S1 Dataset) associated with plant transpiration and growth; calculation of RH is based on the dry and wet adiabatic lapse rate changes in vertical air temperature and stabilisation of RH at 100% once the air reaches saturation; RH decreases as the saturated air subsides
**Wind velocity** (m s^-1^) **and direction** (° from true North)	Modelled according to the full 3D Navier-Stokes equations, incorporating the effects of atmospheric turbulence and thermal processes [29]. Model calculations are based on a boundary-fitted coordinate system. Initial boundary conditions are specified by the growing-season surface mean Ta and wind velocity and direction determined from data collected at the Noushahr climate station (Fig 2), and (ii) an assumed upper wind velocity of 6 m s^-1^ at 500 m. Atmospheric temperature stratification is assumed neutral, a common state of the planetary boundary layer under windy daytime conditions [30]	Wind velocities (Fig 3) can have both positive and negative consequences on plant growth, both from a physiological and mechanical-disruption point of view [31–33]
**Surface reflectance**, bands 1 through 7 (non-dimensional)	Landsat-8 30-mresolution multi-spectral imagery (http://earthexplorer.usgs.gov/, last accessed on June 2014); corrected by the black-object subtraction method [34]	Surface reflectance differs between plants of different species [1]
**Surface radiance**, bands 10 and 11 (W m^-2^ srad^-1^ μm^-1^)	Landsat-8 30-mresolution multi-spectral imagery (http://earthexplorer.usgs.gov/, last accessed on June 2014)	Thermal infrared emissions can be used to describe variation in land surface temperatures, as well as assist in the differentiation of plant species [1]

**Fig 1:**
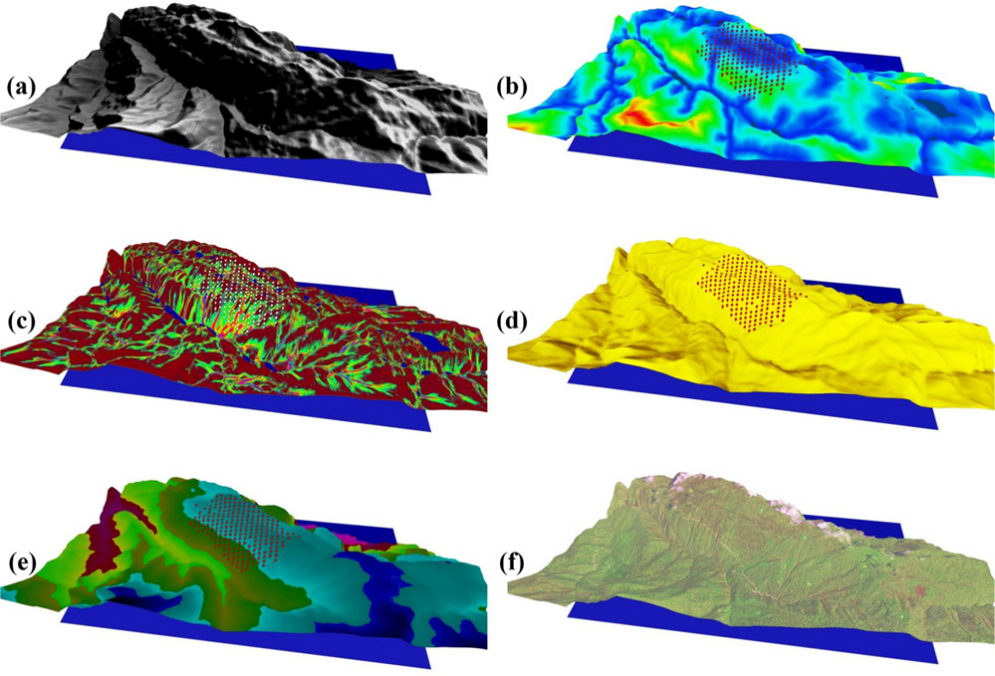
Modelled biophysical surfaces. (a) elevation (m AMSL), (b) ground height above nearest drainage point (HNDP, in m), (c) topographic wetness index (non-dimensional), (d) growing-season accumulated cloud-free insolation (MJ m^-2^), (e) mean growing-season air temperature (°C), and (f) Landsat image of the study area. Variation in colour corresponds to variation in the various variables.

All surfaces are calculated or resampled at 10-m resolution; illustrations of some of these surfaces are provided in Figs. 1 and 3.

**Fig 2:**
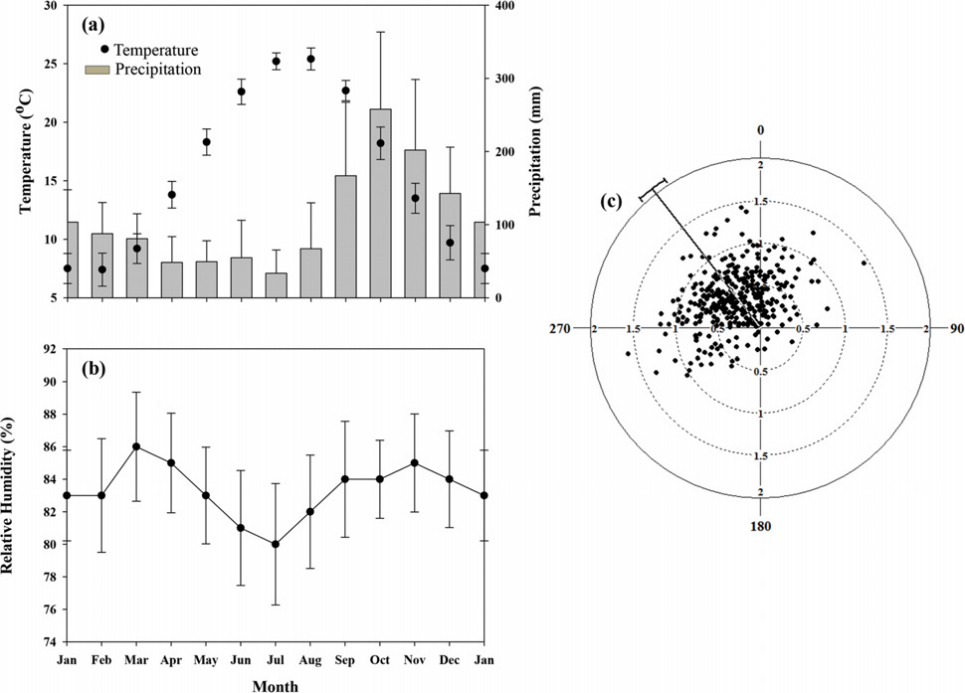
Climatological summaries for Noushahr station. (a) air temperature (°C), precipitation (mm), (b) relative humidity (%), and (c) wind velocity (m s^-1^) and direction (° from true north) based on climate data recorded from 1977 through 2005. The bars in all instances represent the level of data dispersion for individual variables. Mean annual wind direction is 322.1° (±47.9°, standard deviation) from true north; its value varies to 332.6° (±37.5°) during the growing period. Near-surface growing-period average wind velocity is about 0.64 m s^-1^ (±0.28 m s^-1^).

**Fig 3:**
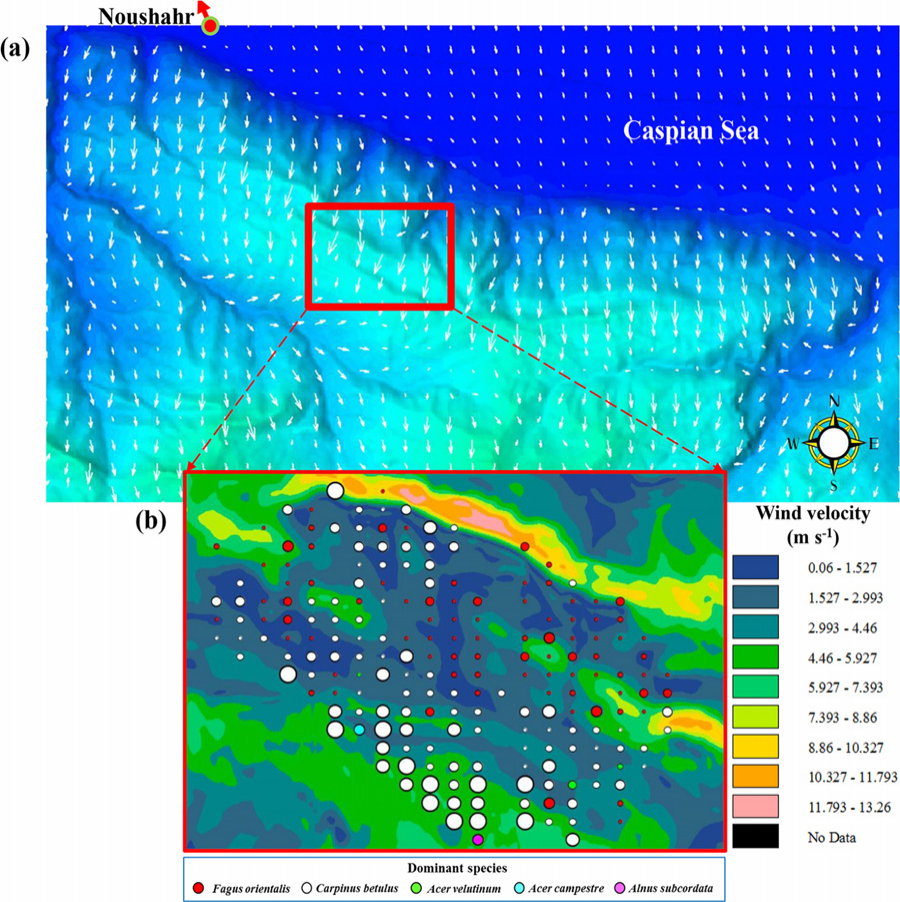
Modelled wind. (a) direction (° from true north) and (b) velocity (m s^-1^; background colours) for the study area. Coloured circles in (b), representing individual plots, vary in size according to observed tree species richness; large circles represent plots with high tree species richness (e.g., SR = 7 species per 0.1-ha plot) and small circles, low species richness (e.g., SR = 1 species per 0.1-ha plot). Plot tree-species dominance (accounting for ≥ 33% of all trees in a plot) is labelled by colour.

## Materials and methods

### Ethics Statement

No specific permission was required to conduct field research in the university forests, where scientific research by university personnel is encouraged. Our field sampling did not involve the destructive sampling of either endangered or protected forest species.

### Study area and plot network

**Fig 4:**
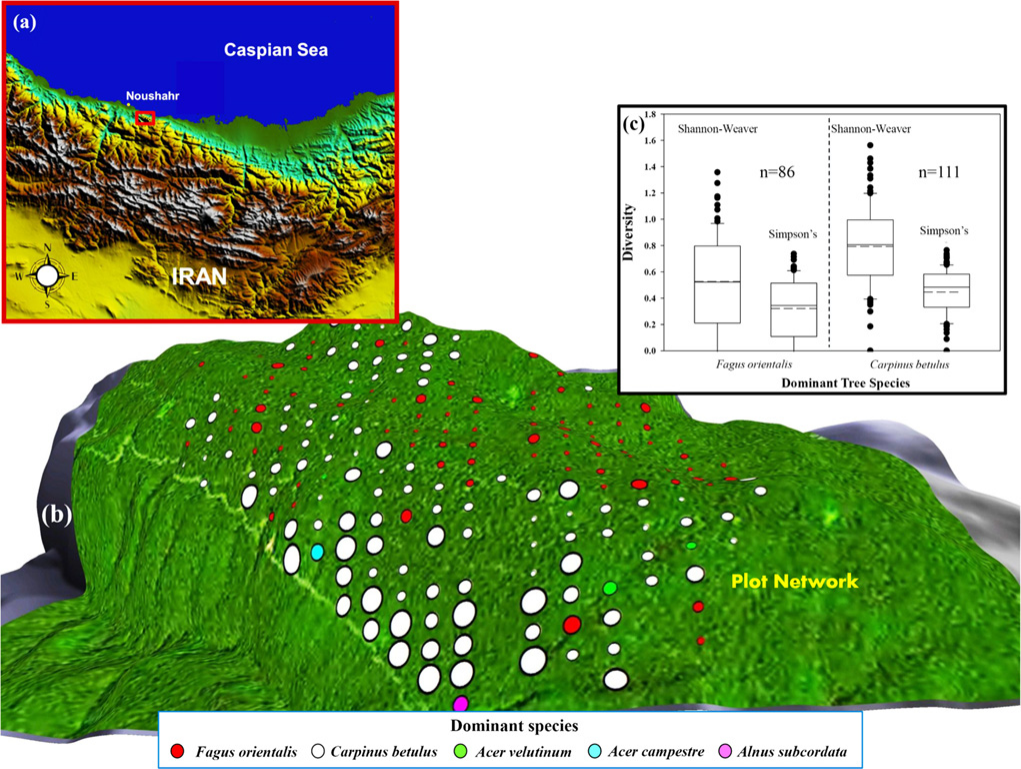
Study site in northern Iran. (a) inset map and (b) network of forest-inventory plots central to the study. Location of the port city of Noushahr in the inset map is indicated next to the plot network. Coloured circles in (b), representing individual plots, vary in size according to observed tree species richness; large circles represent plots with high tree species richness (e.g., SR = 7 species per 0.1-ha plot) and small circles, low species richness (e.g., SR = 1 species per 0.1-ha plot). Plot tree-species dominance (accounting for ≥ 33% of all trees in a plot) is labelled by colour. Boxplots in (c) give species diversity as a function of the Shannon-Weaver and Simpson’s index for *Fagus orientalis*- and *Carpinus betulus*-dominated plots, covering 97.5% of all plots considered (197 of 202 plots). The remaining plots are dominated by *Acer velutinum* (3 plots), *Acer campestre* (1 plot), and *Alnus subcordata* (1 plot). The median and mean of individual distributions are indicated by the solid and dashed lines within the boxes. The 25th and 75th percentile of the data are given as the lower and upper boundaries of the box; the 10th and 90th percentile are given at the lower and upper limits of the whiskers. Values smaller or larger than the 10th and 90th percentile are indicated as black circles.

In northern Iran, along the southwestern coast of the Caspian Sea (Fig 4a), the Hyrcanian forest covers approximately 50,000 km2, including the provinces of Gilan, Mazandaran, and Golestan. Due to its humid sub-Mediterranean climate (Fig 2) and fertile soils, this region is renowned for its high forest productivity [35,36]. Intensive human settlement in the lower elevations, as early as 1,100 AD, has left large portions of the lowlands void of forest cover [36].

Oriental beech (*Fagus orientalis Lipsky*) forests in this region are mixed with *Carpinus betulus*, *Alnus subcordata*, *Acer velutinum*, and several other tree species and shrubs [36]. These forests are mostly broadleaved, but *Taxus bacata* and *Cupressus* species do appear on some specialised sites [37]. Close-to-nature silviculture (a management system based on small-scale interference and group selection) is the harvesting method currently practiced in the low-lying areas of the greater Hyrcanian forest. This forest-management approach is best suited for establishing and maintaining mixed forests and permanent forest cover [37,38].

The experimental forest (Kheyrud Forest of the University of Tehran) is an 80-km2 unmanaged constituency of the greater Hyrcanian forest located about 7-km east of the port city of Noushahr (36° 39' N, 51° 30' E; 7.5 m above mean sea level, AMSL; Fig 4a). The northern lower boundary of the Gorazbon section, one of eight sections of the Kheyrud Forest and location of the plot network (Fig 4a and 4b), sits at 1,010 m AMSL; the section’s highest elevation lies at about 1,380 m AMSL. The Kheyrud Forest consists of 80 different tree species and 50 shrub species. Mean annual precipitation in the area is about 1,397 mm (758 mm, during the growing season) based on climate data from 1977–2005, with October and July, respectively, being the wettest (258 mm) and driest month (33 mm) of the year (Fig 2a). Annual ${\bar{\text{T}}}_{a}$ is 15.5°C, with February and August, respectively, being the coldest (7.4°C) and warmest months (25.4°C; Fig 2a). Mean annual pan evaporation at Noushahr is about 1,031 mm, with highest average monthly evaporation occurring in August (155 mm) and lowest, in January (26 mm). Local relative humidity exceeds 75% for most of the year (Fig 2b). Along the southwestern coast of the Caspian Sea, wind velocity and direction from true north (N) during the growing season averages about 0.64 m s^-1^ and 332.6° (Fig 2c). Following the soil-taxonomic system of the USA Department of Agriculture, soils in the area are classified as highly productive udic alfisols.

The plot network in the Gorazbon section is designed on a rectangular grid (150 m × 200 m) and consists of 258 fixed-area circular plots of 0.1 ha each (Fig 4b; [39]). Tree species richness was determined at each plot from basic tree species identification and tallying. Prominent tree species in plots include *Fagus orientalis*, *Carpinus betulus*, *Acer velutinum*, *Acer campestre*, *Alnus subcordata*, *Quercus castaneifolia*, *Parrotia persica*, *Tillia begonifolia*, and *Ulmus glabra*. Total number of plots available for the current analysis was 202; many of the unused plots had missing site information, including GPS (global positioning system) coordinates, preventing their geo-referencing.

### Development of numerical surfaces

Fundamental to the spatial calculation of abiotic surfaces or their surrogates at mid-resolution is the DTM of the Gorazbon section (Fig 1a). DTM-height data is derived from the Advanced Spaceborne Thermal Emission and Reflection Radiometer (ASTER) 30-m resolution Global Digital Elevation Model v. 2 (GDEM; http://asterweb.jpl.nasa.gov/gdem.asp, last accessed on June 2014). Descriptions of the various abiotic and associated surfaces, including their proxies and their derivation, can be found in Table 1. Values of abiotic and proxy variables at forest-plot locations were summarised separately as averages of values falling within each individual 0.1-ha plot (Fig 4b).

### Combining HNDP and TWI as a proxy of soil water content

Tree species differ in their soil water requirements and tolerances [40]. When soil water content (SWC) is limited or at saturation levels for extended periods of time, photosynthesis and tree growth is reduced to a level consistent with the trees’ tolerance of existing conditions. Optimal SWC allows for more efficient utilisation of soil nutrients [41], allowing for increased biochemical reactions, biomass production, and potentially, species distribution [42].

As soil information and precipitation patterns for the study area were not available, we opted to represent soil water distribution as a function of HNDP and TWI. Combining the two variables (Fig 1b and 1c; Table 1) in a single representation of SWC is reasonable as HNDP approximates the influence on SWC relative to the vertical distance to drainage, particularly in proximity to drainage channels, whereas TWI approximates the influence relative to the flow characteristics of the terrain (i.e., pathways and pooling locations). Together, HNDP and TWI have been assessed to improve description of surface-water-flow-related processes and features in forested landscapes [11].

### Insolation

Available solar radiation (direct + diffused) alters tree growth and tree distribution differently for different species [3]. Shade-intolerant species, such as white birch (*Betula papyrifera*) and red maple (*Acer rubrum*), exploit low light levels less efficiently than shade-tolerant species, like American beech (*Fagus grandifolia*) and sugar maple (*Acer saccharum*; [20]). As a result, shade-intolerant species tend to have lower growing potentials in areas intrinsically low in sunlight. Sensitivity of seedlings and saplings to differences in insolation in some hardwood species, such as in the beeches, changes as the plants mature ([43]; Table 2).

**Table 2:** Life traits of dominant tree species in 97.5% of sampling plots; a compilation of internet sources, e.g., Flora ii. in Persia webpage (consult www.iranicaonline.org/articles/flora-ii-in-persia, last accessed on June 2014), and Tabari et al. (2007) and Heshmati (2007) [44,45].

Species	Elevation (m AMSL)	Light Requirement	Soil Moisture	Comments
*Fagus orientalis*	Part of the cold-deciduous montane forests; can be found growing between 700–2,000; naturally-growing dense stands are found at 1,000–2,000 and the better stands at 900–1,500	Tolerant of heavy shade, while young	Need a well-drained soil and regular wetting; large trees can withstand the occasional drought; saplings are more resistant to drought	Wind pollinated; late frost, early heavy snow, and direct sunlight can damage saplings
*Carpinus betulus*	Part of the cold-deciduous lowland forests; can occur in greater numbers at lower elevations; can also be found growing at higher elevations between 700–1,800	Full sunlight to partial shade; warm climate	Occasionally moist, well-drained soils; tolerant to drought	Seed dispersal by wind currents

### Air Temperature

Plant metabolism, growth, and SR are impacted by temperature [3]. For this reason, plant distributions can correlate fairly well to indices of annual heat inputs. In this study, we use the growing-season ${\bar{\text{T}}}_{a}$ as an index of long term growing-period heat input (Table 1).

### Relative humidity

Relative humidity directly influences the water relations of plants [46] and indirectly affects leaf growth, photosynthesis, pollination, and biomass production [47]. In view of the fact that there were only a few climate stations recording RH (all in low-lying areas), maps of RH were developed on well-known meteorological principles associated with the orographic displacement of moist air [12]. Long term DTM-based calculations of RH proceeded in a manner analogous to that described in S1 Appendix, in relating spatial variation in RH to changes in topography in the direction of the prevailing wind (N-NNE; based on model projections, see Fig 3a) and low-elevation growing-season ${\bar{\text{T}}}_{a}$ and RH prior to the lifting of the air [i.e., 21.2°C and 83%, respectively (Fig 2)].

### Wind

An equally important environmental variable affecting plant production and potentially species presence is wind velocity [20,33]. Wind is not usually considered in SR-studies, because of the difficulty in estimating its velocity and direction spatially. Here, we use a computational fluid dynamics simulator [29] to model wind flow over complex terrain (Fig 3) characterised by the study area’s DTM; consult Table 1, for additional information concerning modelling of surface wind.

### Multi-band surface reflectance and radiance

Different tree species respond differently to energy from the electromagnetic spectrum [1]. As a result, RS data at the appropriate spectral resolution should, in principle, have a part in detecting differences in trees of different species. However, the existing literature remains ambiguous as to which spectral bands are the most suitable in this effort [1], above all in high-elevation forests of northern Iran. Consequently, to address this ambiguity, we investigate the extent SR can be modelled by surface reflectances (non-dimensional, bands 1 through 7) and surface radiances (W m^-2^ srad^-1^ μm^-1^, bands 10 and 11) acquired from a Landsat-8 multi-spectral image of the study area (at 30-m resolution), taken on 19 June, 2013, at 10:46:13.22 local time (WRS, Path 165 and Row 35; courtesy of the USA Geological Survey, 2014).

### Relating plot-estimates of environmental variables to Species Richness

Symbolic regression, or symbolic function identification, is used to determine from the list of independent variables in Table 1, which site variables are particularly crucial in explaining spatial variability in SR. Symbolic regression is a procedure founded on evolutionary computation in searching for algebraic equations, while reducing the difference between target values and values calculated with equations generated with the procedure [48]. Different from conventional regression techniques that determine parameters of known equations, no specific mathematical expression is needed as a starting point to the approach. Rather, primary expressions are formed by randomly combining primitive base functions of input variables (linear or otherwise) with algebraic operators. Equations retained by the procedure are those that replicate the target output data better than others; undesirable solutions are rejected. The procedure stops whenever the desired accuracy in data replication has been reached. In order to balance the relative contribution of each plot-estimate of SR in the development of a generalised expression of SR, SR-values were weighted as a function of the inverse of their occurrence (i.e., number of times it occurs) in the dataset. This was done to ensure that values that are not commonly observed (e.g., SR = 7 species per 0.1 ha plot) contribute as much to the explanation of SR as values that are more frequently observed (e.g., SR = 2–4 species per 0.1 ha plot).

## Results

### Species dominance vs. environmental factors

**Fig 5:**
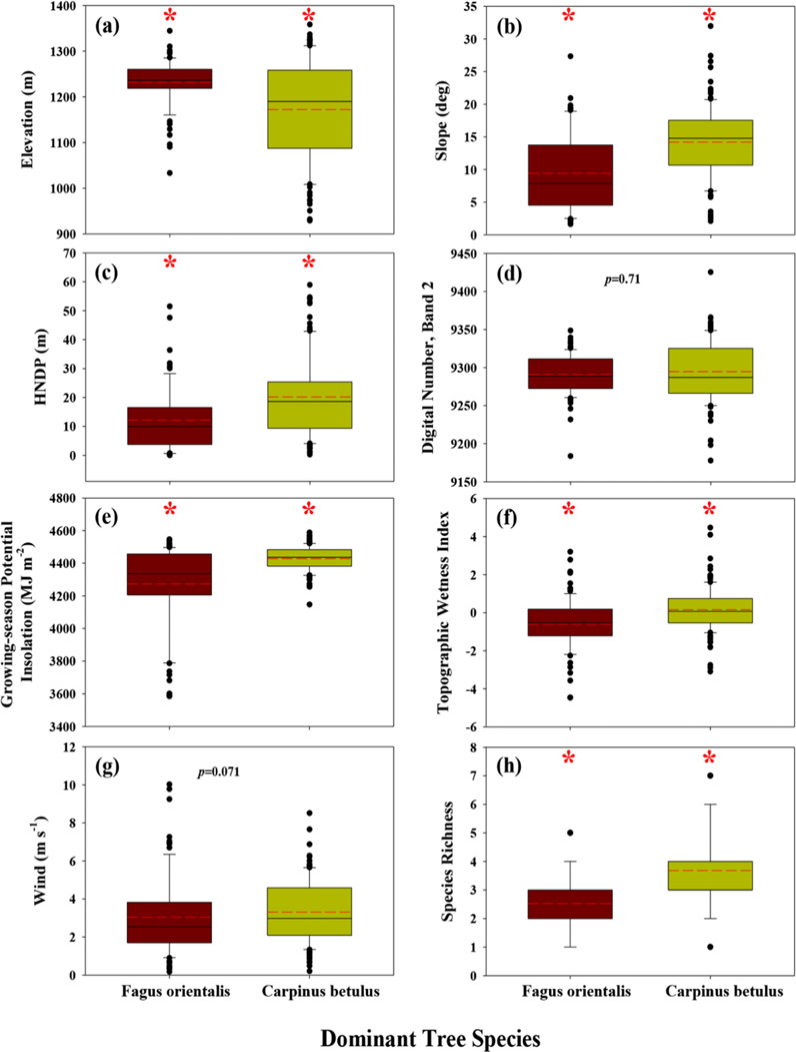
Sampling-plot averages of site and environmental values for *Fagus orientalis*- and *Carpinus betulus*-dominated plots. All distributions were statistically different from normal, based on Shapiro-Wilk normality tests and *p*-values < 0.05. Plots labelled by red asterisks identify statistically-significant differences between medians (based on rank-sum tests and *p*-values < 0.001) for values associated with *Fagus orientalis*- and *Carpinus betulus*-dominated plots.

Fig 5 displays distribution plots for selected variables (listed in Table 1) for both *Fagus orientalis*- and *Carpinus betulus*-dominated forest plots (involving ≥ 33% of all trees in a plot).

In general, *Fagus orientalis*- and *Carpinus betulus*-dominated plots occupy a different part of the area’s landscape. The *Fagus orientalis*-dominated plots occur mostly on the flatter, higher elevation, and wetter and potentially cooler parts of the landscape (median slope, elevation, and HNDP of 7.9o, 1236 m AMSL, and 9.9 m, respectively), whereas *Carpinus betulus*-dominated plots occur on the higher-sloped, mid-elevation, and potentially dryer and warmer parts of the landscape (median slope, elevation, and HNDP of 14.8o, 1190 m AMSL, 18.6 m, respectively; Figs. 4 and 5a and 5b and 5c; *p*-values < 0.001). Due to their improved position with respect to the sun’s energy, *Carpinus betulus*-dominated plots are likely beneficiaries of higher insolation during the growing period than their *Fagus orientalis*-dominated counterparts. Median growing-season cloud-free insolation for *Carpinus betulus*-dominated plots is estimated at about 4436.5 MJ m^-2^, compared with 4336.1 MJ m^-2^ for *Fagus orientalis*-dominated plots (Figs. 1 and 5e; *p*-value < 0.001). At higher elevations, where *Fagus orientalis* is common (Fig 4), we can expect insolation to be further reduced as a result of increased cloud cover resulting from localised orographic lifting of moist air and associated cloud-forming processes. In general, species diversity and SR are greatest in *Carpinus betulus*-dominated plots (Figs. 4c and 5h). There is no statistical difference in the median of wind velocity and surface reflectance of blue light (Landsat-8, band 2) between *Fagus orientalis*- and *Carpinus betulus*-dominated plots (Fig 5d and 5g; *p*-values > 0.05), suggesting near-uniform variation in these two variables, when examined across all plots.

### Tree species richness vs. environmental factors

It was shown with reasonable accuracy (mean-absolute and root-mean-squared error of 0.50 and 0.78) that only four variables were needed to explain 85% of observed plot-level variation in SR (with a coefficient of determination, r2, of 0.85), namely in order of declining influence: (i) wind velocity (accounting for 51% of the total variation in SR); (ii) surface reflectance of blue light; (iii) HNDP; and (iv) TWI. Individual contributions to the explanation of SR are provided in Table 3.

**Table 3:** Relative contribution of wind velocity (WND; m s^-1^), surface reflectance of blue light (B2; non-dimensional), ground height above nearest drainage point (HNDP; m), and topographic wetness index (TWI; non-dimensional) in a linear description of spatial variation in tree species richness (number of trees per 0.1-ha plot).

		Independent Variable				Individual contribution (%)	
Solution	r^2 a^	WND	B_2_	HNDP	TWI		
1	0.512	×				WND	51.2
2	0.626	×	×			B2	11.4
3	0.708	×	×	×		HNDP	8.2
4	0.775	×	×	×	×	TWI	6.7
5	0.849	×	×	×	×	Linear combinations + Thresholds	7.4

“×” marks the variables included in the various equations generated with symbolic regression. Solutions 1–4 are based on linear combinations of variables identified in the Table. Solution 5 is similar to solution 4, except solution 5 incorporates thresholds with respect to the portion of HNDP and TWI that best describes the area’s distribution of soil water content. The unused variables in Table 1 had little to no role in explaining the variation in species richness.

^a^ r^2^ is based on a plot-level comparison of modelled and corresponding field-based estimates of SR.

The equation that best described SR was:

$$\begin{array}{l} \;S\text{R}=round\left[0.598WND+768.356B_{2}+0.513\min\left(0.1HNDP-1.657,0.584\right)+a_{1}+a_{2}-70.112\right],\\ where\;a_{1}=\{\begin{array}{c} 1;\text{ if}\left[\left(\text{TWI-0.0644}\right)>0.514\right]\\ 0;\text{ otherwise} \end{array}\;,\\ \;a_{2}=\{\begin{array}{c} 1;\text{ if}\left[\text{tan}\left(\text{19.500}\cdot \text{WND-55.643}\right)<\left(2.789+TWI-WND\right)\right]\\ 0;\text{ otherwise} \end{array},\;and \end{array}$$ (1)

WND and B2 are the near-surface wind velocity (m s^-1^) and Landsat-8-acquired surface reflectance in band 2 (non-dimensional). The “round()” function in equation (1) converts real numbers to whole numbers, rounded to the nearest value. The other variables in Table 1 had little to no effect on the final solution (results not shown).

Interaction of HNDP and TWI in equation (1) is addressed through terms three and four of the main equation. In general, when HNDP = 0 (in vicinity to open water), the effect is to decrease SR by 0.85 units, or one species per 0.1 ha plot when rounded to the nearest whole number. At 0 < HNDP ≤ 16.57 m, the effect is to lessen the reductions in SR, until SR is no longer affected by changes in HNDP [i.e., the third term of equation (1) becomes progressively less negative, until it reaches zero at HNDP = 16.57 m], and when 16.57 < HNDP ≤ 22.41 m, increments in SR gradually increase (become progressively larger), until HNDP = 22.41 m, where increments stabilise at 0.3 units. The effect of HNDP on SR remains unchanged for all HNDP-values > 22.41 m; the influence of HNDP on SR stays constant at 0.3 units, which when rounded to the nearest number becomes zero.

**Fig 6:**
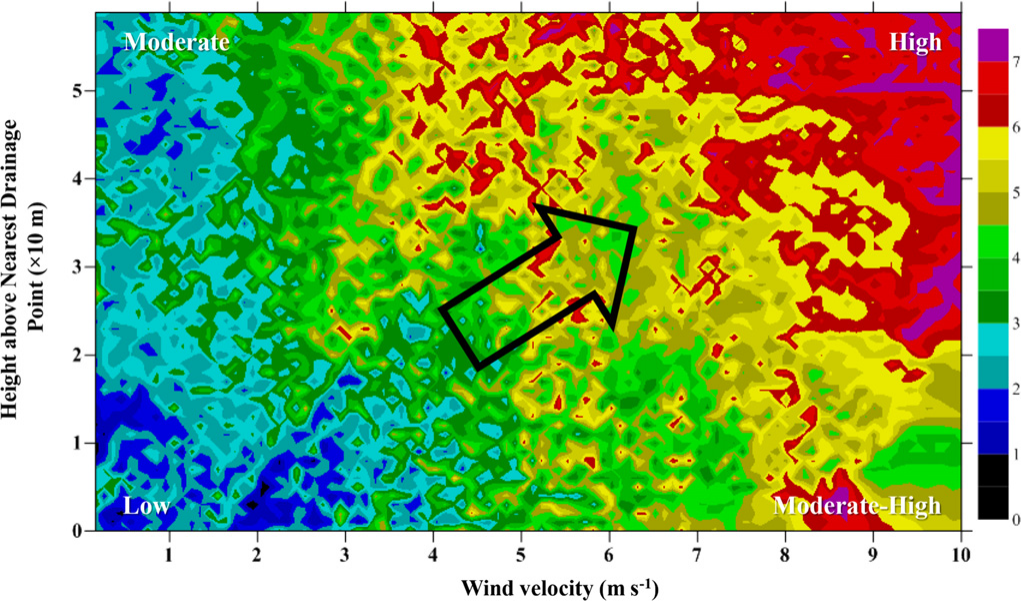
Contour plot of tree species richness as a function of wind velocity (m s^-1^; x-axis) and ground height above nearest drainage point (HNDP, m; y-axis). The arrow in the centre denotes the general increase in tree species richness as wind velocity and HNDP increase linearly simultaneously.

**Fig 7:**
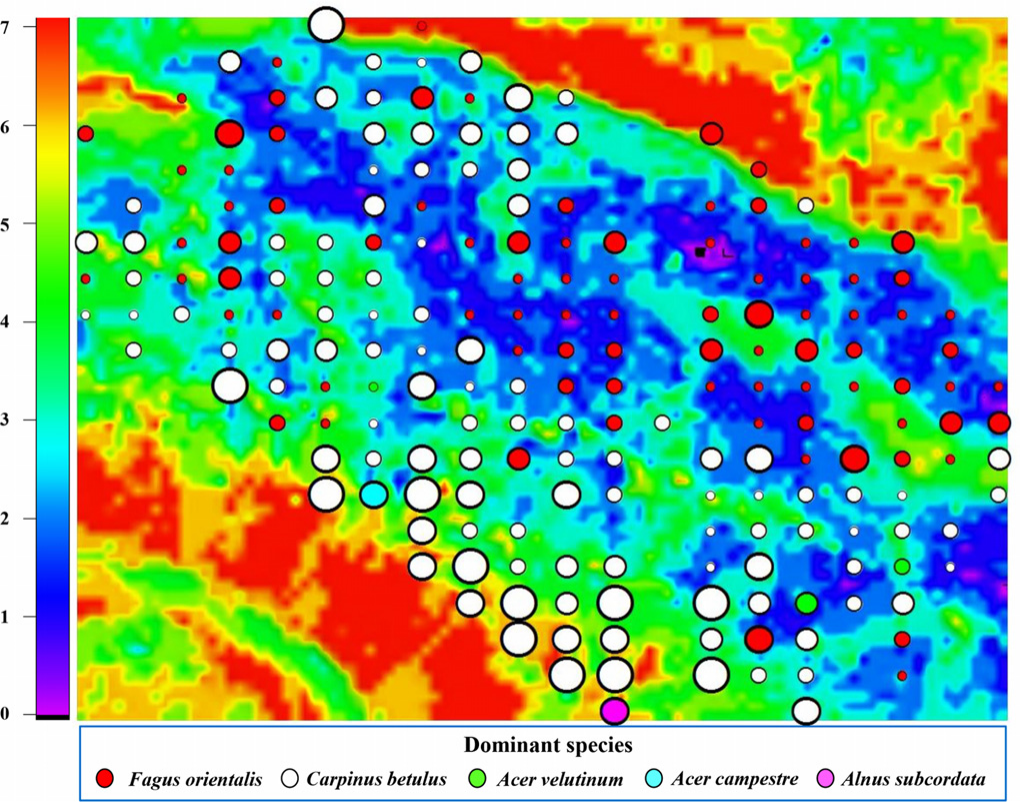
Mapped tree species richness derived with equation (1). Coloured circles, representing individual plots, vary in size according to observed tree species richness; large circles represent plots with high tree species richness (e.g., SR = 7 species per 0.1-ha plot) and small circles, low species richness (e.g., SR = 1 species per 0.1-ha plot). Plot tree-species dominance (accounting for ≥ 33% of all trees in a plot) is labelled according to colour (Fig. 4).

TWI operates as a correction and only becomes effective when TWI ≥ 0.578 [the fourth term in equation (1), i.e., a1 is set to one]; when conditions are not met (i.e., TWI < 0.578), there is no net change in SR as a result of TWI [i.e., a1 in equation (1) is zero], and the representation of SWC is based exclusively on values of HNDP. The second correction to SR is based on a comparison of a linear function incorporating both the effects of wind velocity and TWI with the tangent of a linear function of wind velocity. The three-way interaction of wind velocity, HNDP, and SR is demonstrated in Fig 6. Clearly, zones of the landscape exposed to high wind velocities and HNDP’s (Figs. 1b and 3b) tend to promote high SR; the opposite is true, when wind velocities and HNDP’s are both very low. Fig. 7 summarises the results with respect to calculated SR (background colours) and field-based estimates (coloured circles).

## Discussion

Numerical estimates of abiotic conditions and their proxies (i.e., wind velocity, HNDP, and TWI) at the plot level, along with satellite-acquired surface reflectance of blue light, can explain a significant portion of plot-level variability in SR in the region’s high-elevation forests (~85%). A significant portion of the unaddressed variation could possibly have been accounted for by including soil-related information (currently unavailable), including soil nutrient content [3], in the evaluation of SR. It is interesting to note, that numerically-based biophysical surfaces of a few abiotic variables or their proxies at 10-m resolution are sufficiently sophisticated to model plot-SR (within 0.1 ha) at reasonable levels of accuracy (r2 = 0.85; consult Table 4, for results of comparable studies), despite being generated from a base DTM with an effective spatial resolution of 30 m.

**Table 4:** Partial literature review of plant species richness (SR) as a function of environmental heterogeneity; area and scale of application, methods, and results.

Application Area & Scale	Approach	Results	Source
Majella National Park, Italy; mesoscale, 170 km2	The study assessed the accuracy of detecting SR in a forest site by combining mid-resolution images from satellite with environmental data of elevation, slope, aspect, and solar radiation in an artificial neural network classifier	Map accuracies obtained for Landsat-TM and ALOS images were 60 and 53%, respectively. Use of environmental data increased accuracies to 91 and 81%	[2]
Kevo Nature Reserve, north Finland; mesoscale, 362 1-km grid squares	Using generalised linear modelling, multiple regression models of SR were built with a training set of 257 grid squares and 33 environmental variables	The fitted model explained 51% of the variation in total number of vascular plant taxa in the testing set; altitudinal variables were the better predictors of SR	[9]
Northeast Iberian Peninsula; mesoscale, 100 km2 grid	Relationship between SR and environmental variables was tested by a weighted analysis of variance using generalised linear models	Environmental heterogeneity addressed 67% of the spatial variation in SR	[49]
Tutuila, American Samoa, South Pacific Ocean; mesoscale, 142 km2 grid	Expressed SR along an elevational gradient from a mountain ridge, mid-slope, to valley position	Chi-square analysis indicated that nine tree species (of a total of 52 species) had strong preference for specific topographic position	[50]
Two tropical forest sites: (1) a tropical montane cloud forest and (2) a lowland Amazonian forest; microscale, ten 25 m × 25 m plots per forest region	Examined the micro-scale variability in tree SR through a combination of ground-based plot studies and computer-based analyses of 16 terrain characteristics	SR was found to correlate fairly well with slope mean curvature, with high SR found on convex slopes (r2 = 0.73)	[51]
Cloud forest in Monte de Neblina de Cuyas, northern Peruvian Andes; microscale; study site was situated at altitudes ranging from 2,359–3,012 m AMSL, mostly on a southwest-facing slope	Examined species-habitat associations in three 1-ha plots using the torus-translation method	When topographic and forest structure variables were combined in the definition of habitat, SR with significant plant associations (*p* < 0.05) was higher (in 15 species) than when either topographic (nine species) or forest structure variables (five species) were used independently	[52]

Wind velocities can have both positive and negative consequences on plants, both from a physiological or mechanical perspective. In low wind velocities, large boundary-layer resistances between the air and leaf surface impede the transfer of carbon dioxide (CO2) and water vapour to and from the plants [53], causing growth in plants to proceed at reduced rates [31–33]. In high wind velocities, wind distorts the growing pattern of plants by: (i) applying constant bending-pressure on the main stem and/or its parts (branches, twigs, leaves, etc.), leading to breakage and, in some high velocity events, uprooting of entire trees; and (ii) contributing to the rapid transfer of water vapour from the plants to the atmosphere and triggering closure of the stomata to prevent desiccation and, in the process, reducing the uptake of CO2 and, consequently, reducing plant growth [32]. Seedlings and saplings on wind-exposed terrain can expire from the drying effects of wind, particularly in moderate to high velocity events [20]. However, high RH’s of the study area (Fig. 2b) may help lessen the threat of desiccation. Most favourable growing conditions with respect to wind velocity have been demonstrated to occur somewhere in between these two extremes [31]. In the current study, wind velocity of ~2.4 m s^-1^ corresponds with optimal growing conditions for *Fagus orientalis*. Chronic physical disturbance of forests by high velocity events along wind-exposed terrain is thought to increase spatial heterogeneity and, thus, promote species proliferation in affected areas [20,54]. At our study site, SR-patterns are consistent with a wind-dominated disturbance regime (Fig. 3b). Historical wind velocities are shown to decline more rapidly at higher elevations than at lower elevations as a result of global climate change [55]. This has the potential to impact biophysical processes (e.g., seed dispersal, evapotranspiration, and disturbance regime) and SR in high-elevation forests of northern Iran.

Overlain on the wind-disturbance regime is the effect of SWC on SR, demonstrated through the combined influence of HNDP and TWI, as addressed earlier. Its overall impact on SR is small compared to the effect of wind (15% vs. 51%; Table 3), but strong enough to be a viable factor in driving local variation in SR (Fig. 7). Zones of less than optimal HNDP’s (~0–2 m), indicative of high SWC, and low wind velocities tend to support low SR (Fig. 6) in *Fagus orientalis*-dominated plots (Fig. 7) at the exclusion of other tree species. Greatest soil moisture is expected to be found in landscape depressions (vernal pools; light purple areas at the centre of Fig. 7) that regularly fill up with water, particularly during the snowmelt season of the year. Presence of *Fagus orientalis* in the wetter part of the landscape is in keeping with *Fagus orientalis*’ preference for intermittent wetting of the soil (Table 2). *Fagus orientalis* growing in areas of the landscape that remain wet for a significant part of the growing season, tend to grow very slowly, i.e., ≤ 1.3 cm over a 9-year period, based on diameter at breast height measurements taken locally in 2003 and 2012. Trends in SR in *Acer velutinum*-, *Acer campestre*-, and *Alnus subcordata*-dominated plots tend to follow those observed in *Fagus orientalis*- and *Carpinus betulus*-dominated plots (Figs. 3b and 7).

Variation in surface reflectance in the Landsat-8 blue band (band 2) contributes towards explaining 11.4% of the variation in SR (Table 3). Blue light is absorbed by chlorophyll and, as a result, affects the amount of blue light reflected back to the RS sensor. Maximum between-species variation has been identified by others to occur in the visible range, namely between 0.45–0.70 μm [1]. Some studies have indicated that light at the blue edge of the green peak may be more useful in species separation than light at the red edge [1]. However, blue light is sensitive to the state of the atmosphere (i.e., concentration of aerosols, dust, etc.) and thus the ability of reflected blue light to sense different species has been questioned [1]. Most likely the uniform atmospheric conditions of the area, due to the study area’s small size, has nullified the influence of atmospheric state on the local reflectance of blue light and improved the level to which blue light can differentiate between different tree species.

Although developed for a specified area and time frame, the modelling system can provide the basis for extending the work to larger forested areas and time horizons in scaling-up to entire forested landscapes, while maintaining satisfactory spatial resolution. Because SR is explicitly linked to abiotic conditions of landscapes, the effects of climate change on forest tree SR is potentially quantifiable, providing that rates of change in the various abiotic variables can be estimated from output from existing global or limited-area, regional climate models. This type of work is strongly supported by the need to understand ecosystem relationships as they vary in both space and time, especially in view of current conservation management needs and global climate change [56].

## Conclusions

The current paper presents a semi-empirical approach to the assessment of biophysical and satellite data-based variables in describing local variation in SR in an experimental subsection of the Hyrcanian forest of northern Iran. The approach relates plot-measurements of SR to corresponding plot-values of computer-generated abiotic surfaces (and their proxies, especially in the case of SWC) and Landsat-8-acquired surface reflectance and radiance, with an ability to explain ~85% of the variation in plot-level variation in SR. It is shown by symbolic regression that SR can be largely explained by variation in wind velocity, surface reflectance of blue light, HNDP, and TWI, in order of declining importance. Reduced SR in plots are more frequently observed in areas of the landscape with low wind velocities (< 1.0 m s^-1^ in topographically-sheltered areas) and low HNDP (and potentially elevated SWC), particularly in large, frequently saturated depressions in the flatter parts of the landscape. *Carpinus betulus*-dominated plots are found to occur in the warmer, steeper, and windier parts of the landscape, whereas *Fagus orientalis*-dominated plots tend to occur in the potentially cooler, flatter, and low wind-velocity parts of the landscape. In general, *Carpinus betulus*-dominated plots tend to demonstrate greater tree species diversity and richness, compared to their *Fagus orientalis*-dominated counterparts. Calculations of SR are likely to be improved with the integration of soil inventory data. The methodology presented here (i.e., biophysical-surface development and symbolic regression) is sufficiently robust to address SR-patterns in other forested regions of the world, as long as plot-scale data are available for SR-model development.

## Supporting Information

S1 Appendix Development of relative humidity surfaces. (DOCX)

S1 Dataset Environmental and species data pertinent to the current analysis. (XLSX)
