# Supplementary material for: Landscape Variation in Tree Species Richness in Northern Iran Forests
Source: PLoS One. 2015 Apr 7;10(4):e0121172. doi: 10.1371/journal.pone.0121172 (PMC4388521; doi:10.1371/journal.pone.0121172)
Supplement: S1 Appendix — (DOCX) [file pone.0121172.s001.docx]

**Appendix**

Since few climate stations with the required RH data were available, we produced our own RH surface based on well-known meteorological principles associated with the orographic displacement of moist air [1]. We approached this problem by writing Fortran-90 code that related the spatial pattern of RH according to (i) changes in DTM-defined terrain elevations along a series of transects (or profiles) spaced at 10-m intervals parallel to the direction of the prevailing wind in the experimental forest (i.e., NNE direction, Fig. 4a in main text), and (ii) growing-season $\bar{T}_{a}$ and RH determined at Noushahr prior to the lifting of the air (i.e., 21.2^o^C and 83%, respectively). Initial $\bar{T}_{a}$ and RH along the coast is used to calculate the initial water vapour content of the air in terms of its partial vapour pressure (in hPa) prior being displaced vertically (E_act_ is conserved, until RH reaches 100%), i.e.,

$\left.\text{E}_{\text{act}}\right|_{z}=\left(\text{RH}_{\text{z}}/100\right)\cdot\left.\text{E}_{\text{sat}}\right|_{\text{z}}\text{, and }$ (1)

$\left.\text{E}_{\text{sat}}\right|_{z}=6.11\cdot10^{\left[\text{8.573-}2340/\left(\text{T}_{\text{z}}+273.15\right)\right]},$ (2)

where E_sat_ is the saturation water vapour pressure at temperature T_z_ (in ^o^C; here, z=-21 m AMSL at Noushahr; z increases further inland), and E_act_ is the actual water vapour pressure of the air before and after displacement. To model variability in wind direction along the prevailing NNE direction (i.e., 22.5^o^; with 0 ^o^ being N) we allowed the transect lines to the DTM to be place at angles starting with 0^o^, which were gradually increased to 45^o^ using an angle-increment rotation of 0.1^o^. The movement along each transect and calculation of elevational gradients, T_z_, and RH_z_, potentially of thousands of transects for each increment rotation, is based on Dozier et al. [2] profile algorithm initially used in the calculation of terrain horizon angles. Here, we only need to consider the calculations in the forward direction, as backward calculations in Dozier’s algorithm would suggest a wind direction from SSW.

As the air begins to interact with the topography and ascends, the internal temperature of the air (initially set to $\bar{\text{T}}$ along the southwestern coast of the Caspian Sea) is lowered according to the dry adiabatic lapse rate (i.e., -1^o^C per 100 m [3]), until the air becomes saturated (i.e., RH reaches 100%) at which time the air cools wet adiabatically (~-0.5^o^C per 100 m in rise, due to the release of latent heat with the condensation of water vapour [3]). Further lifting of the air causes excess water vapour to condense and fall out of the rising air, keeping the RH of the rising air constant at 100% (at which time, E_act_ is forced to equate to E_sat_). On descent, the air is compressed and warms dry adiabatically causing the RH to drop. The calculation of RH as the air is displaced both upward and downward is based on a re-arrangement of eq. (1). Final spatial calculation of RH (for individual DTM grid points) entails computing the mean of many RH values calculated for every DTM grid-point generated with each step rotation of the set of parallel transects (from 0-45^o^).

**Reference**

1. Bourque CP-A, Matin MA. Seasonal snow cover in the Qilian Mountains of Northwest China: Its dependence on oasis seasonal evolution and lowland production of water vapour. J Hydrol. 2012;454-455: 141-151.

2. Dozier J, Bruno J, Downey P. A faster solution to the horizon problem. Comp Geosci. 1981;7: 145-151.

3. Lutgens FK, Tarbuck EJ. The atmosphere: An introduction to meteorology. 9^th^ ed. New Jersey: Prentice Hall; 2004.
